# Correction of metal artefacts around orthodontic mini-implants – a micro-CT study in the rat tail model

**DOI:** 10.1038/s41598-025-93411-9

**Published:** 2025-04-01

**Authors:** Robert Kerberger, Giulia Brunello, Nicole Rauch, Dieter Drescher, Bert van Rietbergen, Kathrin Becker

**Affiliations:** 1 Department of Orthodontics and Dentofacial Orthopaedics, Charité-Universitätsmedizin Berlin, Corporate Member of Freie Universität Berlin, Humboldt-Universität zu Berlin, 14197 Berlin, Germany; 2 Department of Oral Surgery, University Hospital Düsseldorf, Moorenstraße 5, 40225 Düsseldorf, Germany; 3 Department of Neurosciences, School of Dentistry, University of Padova, Via Giustiniani 2, Padova, 35128 Italy; 4 Department of Orthodontics, University Hospital Düsseldorf, Moorenstraße 5, 40225 Düsseldorf, Germany; 5 Orthopaedic Biomechanics, Department of Biomedical Engineering, Eindhoven University of Technology, Groene Loper 3, Eindhoven, 5612 AE The Netherlands

**Keywords:** Micro-CT, Peri-implant bone, Peri-implant artefacts, Metal artefact correction, Bone morphometry, Rat tail model, Biomaterials, Medical research

## Abstract

Micro-CT enables volumetric analysis of peri-implant tissue, but grey value alterations due to metal artefacts can impair analyses. This study aimed to assess to which extent peri-implant grey values are affected by metal artefacts at increasing distance to the implant, and whether mathematical correction is possible. In nine rats, two Ti6Al4V orthodontic mini-implants (OMIs), 0.8 mm in diameter and 3.0 mm in length, were placed in a single tail vertebra. Micro-CT scans were performed before (T0) and after (T1) careful removal of the OMIs. Consecutive micro-CT scans were registered and differences in local grey values were computed at increasing distance to the implant (10.4 μm to 405.6 μm). Correction coefficient (CC) computation was performed using a smoothing spline fit, with the distance to the implant and the grey value difference as independent and dependent variable, respectively. To validate the effectiveness of the CC, the amount of calcified bone volume per total volume (BV/TV) was assessed within a standardized volume of interest (VOI) reaching up to 1 mm around the OMIs before and after the application of CC, and the T1-T0 differences between corrected and uncorrected scans were compared using the Wilcoxon signed-rank test. The grey value difference between uncorrected T0 and T1 scans was low in proximity to the implant (32.7%±6.11%) and improved at a distance of at least 100 μm (93.4%±4.46%). CC computation revealed a satisfactory fit (R^2^ = 0.989, RMSE = 0.031) and the difference in grey values was significantly lower after correction (*p* < 0.001). Most VOIs showed significant improvement, though overcorrection was observed in a few cases. Within the limitations of the study, metal artefacts decreased with increasing distance to the OMIs, and significant improvement was possible using the CC.

**Supplementary Information:**

The online version contains supplementary material available at 10.1038/s41598-025-93411-9.

## Introduction

Micro-computed tomography (micro-CT) has been employed for bone microstructural analysis since the late 1980s and its utilization has increased considerably since then^1^. It enables high-resolution, three-dimensional (3D) analysis of trabecular and cortical bone^2,3^. A high agreement has been confirmed through the comparison of micro-CT scans with their corresponding histological samples^4^. Moreover, an additional advantage of micro-CT imaging is its non-destructive nature combined with its capability to provide 3D data^4^ enabling in vivo analyses of specimens^5^.

Despite the aforementioned advantages, a major limitation of peri-implant tissue analyses in micro-CT images are grey value alterations owing to metal artefacts^6–8^, including beam hardening (streaking and cupping), Compton scatter, and blooming artefacts^9^. As a consequence, grey values in reconstructed images may not correspond to the original specimen and grey value alterations may mask the nearby structures or add non-existing ghost elements^10–13^. Due to metal artefacts, bone-to-implant contact (BIC) values, representing the ratio of peri-implant bone volume per tissue volume, tend to be systematically overestimated in micro-CT images but could be partially corrected when measurements were performed by a trained observer^4^. Thus, it might be suspected that an adaption of the grey values in proximity to the implant could have similar effects.

In addition, some authors aimed to perform artefact reduction in micro-CT scans by using filtering of the X-rays, iterative reconstruction algorithms or by performing correction of grey values around highly absorbing tissues in reconstructed images^14–19^. Nonetheless, these approaches also have their limitations, as X-ray filtering obviously cannot be applied after the scan is made while reconstruction algorithms rely on the projection images still being available, which may not always be the case. Most micro-CT suppliers provide dedicated algorithms for artefact reduction. However, these tools are often challenging to customize, and to the best of the authors’ knowledge, there is a quest for straightforward methods to effectively reduce artefacts in the reconstructed images^20^. Moreover, the severity of the artefact will also depend on the implant size and material, and in particular small implants of titanium may not cause major geometrical artefacts but rather influence the grey-level of the voxels.

For the present study, we investigated the effects and correction of artefacts around orthodontic mini-implants (OMIs) made of titanium that were placed in rat tail vertebra, as used in an earlier study^21^. The aims of the present study were: (1) to assess the magnitude to which these peri-implant grey values are affected by metal artefacts, and (2) to investigate if these values can be corrected using a correction coefficient depending on the distance to the implant.

## Results

### Peri-implant grey value comparison, calculation and application of the correction coefficient (CC)

The discrepancy of the grey values between the T0 and T1 scans, i.e. before and after removal of the implants, diminished with increasing distance to the implants (Fig. 1). When analysing the smoothing spline fit, the goodness of fit was high (R^2^ = 0.989 and RMSE = 0.031). At a distance of 10.4 μm, which corresponds to the interface between the first two voxel layers from the implant, the original quotient $\:{q}_{x}$ was 0.33 ± 0.06. This value increased to 0.93 ± 0.05 at a distance of 405.6 μm, corresponding to the interface between voxel layer 39 and 40 from the implant. After $\:CC\left(x\right)$ was applied, $\:{q}_{x}^{{\prime\:}}$ amounted to 1.00 ± 0.20 at a distance of 10.4 μm and 1.03 ± 0.03 at a distance of 405.6 μm (Fig. 1), where values of 1.0 would represent an ideal fit. The Wilcoxon signed-rank test revealed a statistically significant difference between the quotient values before ($\:{q}_{x}$) and after ($\:{q}_{x}^{{\prime\:}}$) the application of $\:CC\left(x\right)$ (*p* < 0.001).

**Fig. 1:**
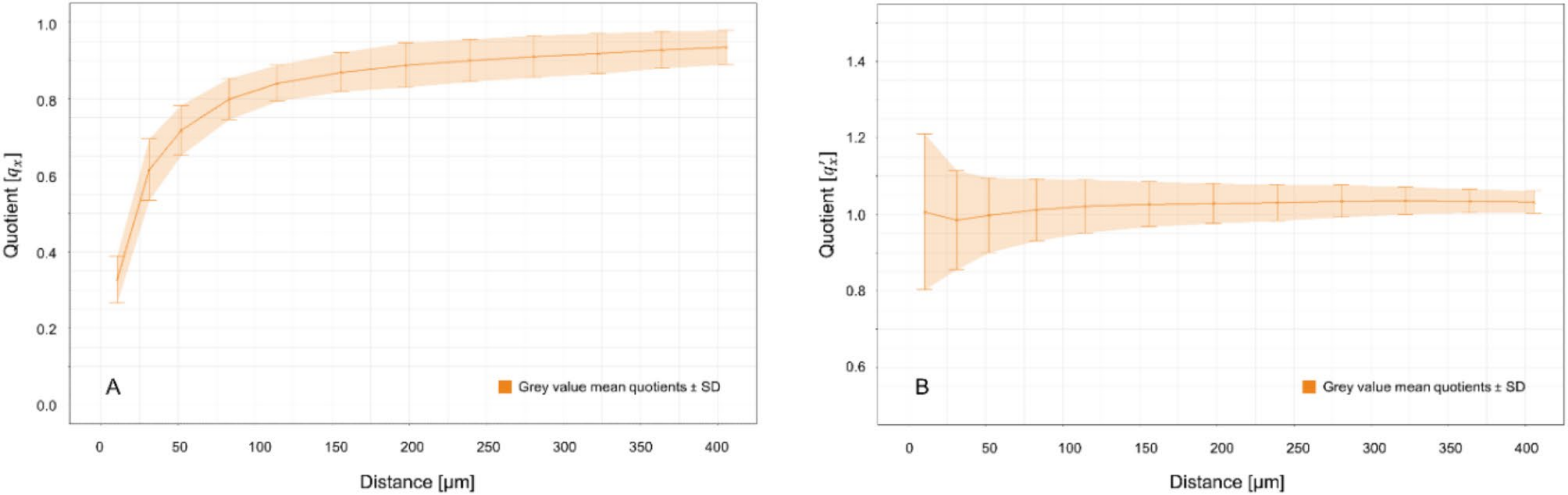
(A) Grey value mean quotient $\:{\text{q}}_{\text{x}}$ ± SD before $\:\text{C}\text{C}\left(\text{x}\right)$ and (B) $\:{\text{q}}_{\text{x}}^{{\prime\:}}$ ± SD after $\:\text{C}\text{C}\left(\text{x}\right)\:$was applied. Both indicated in orange.

### Validation of the CC by BV/TV value comparison

Analysis of the nine defined peri-implant bone regions revealed that after correction residual values ($\:{(BV/TV)}_{T{0}^{{\prime\:}}}-\:{(BV/TV)}_{T1}$) remained above zero in most regions, with values below zero indicating overcorrection observed only in a few proximal and distal regions (i.e. proximal bottom, distal middle and distal bottom), that were less affected by metal artefacts (Fig. 2). In seven regions out of nine, the application of the $\:CC\left(x\right)$ had an impact on the BV/TV calculation; out of these, in five the correction was significant. The strongest influence of metal artefacts was found lateral from the implant (Fig. 2).

**Fig. 2:**
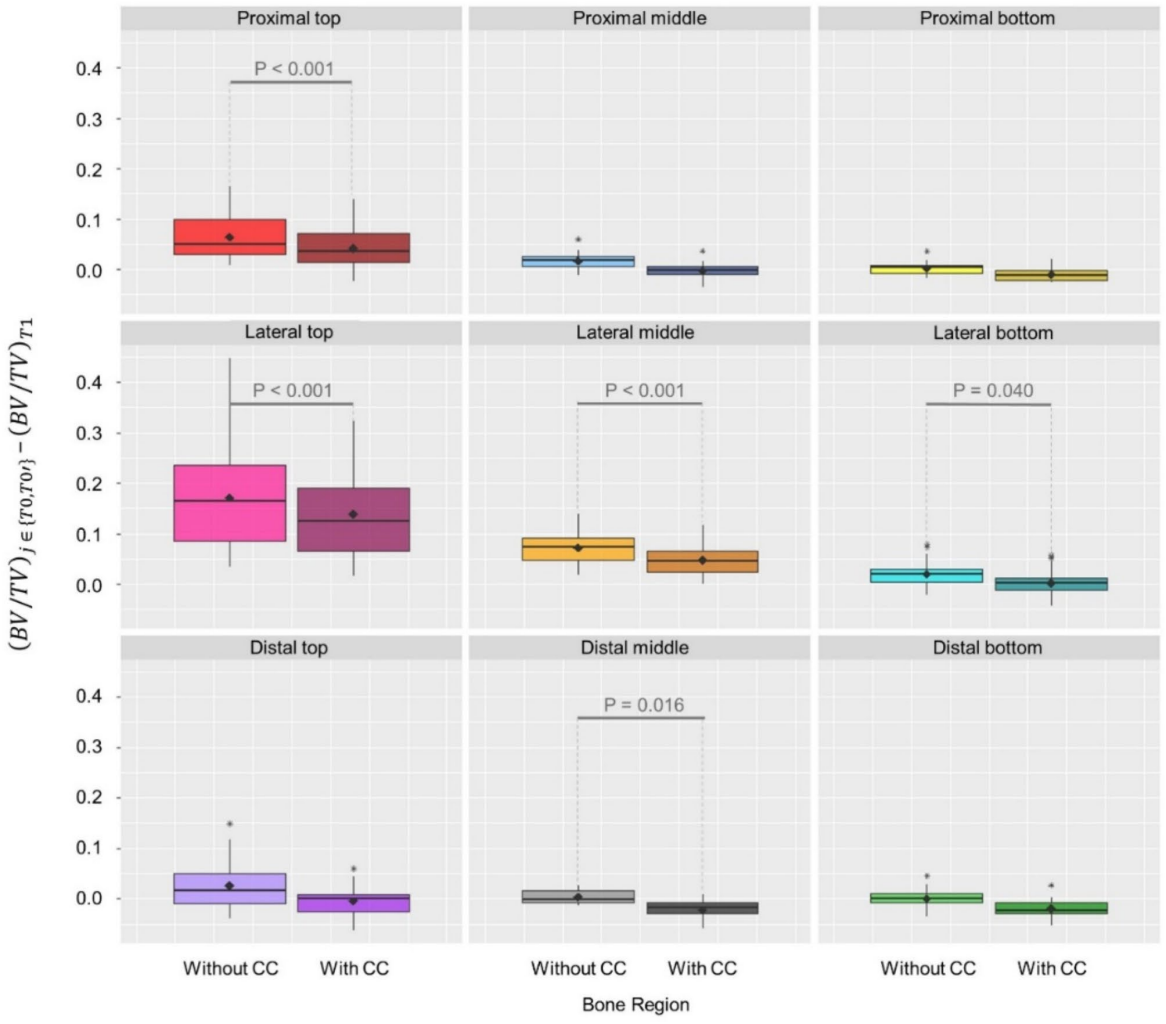
Median $\:\text{B}\text{V}/\text{T}\text{V}$ values + standard deviation and mean values (♦) for the nine segmented peri-implant regions within voxel layer 2–65 from the implant. The amount of peri-implant calcified bone volume per total volume BV/TV in T0 (left boxplot) and the corrected T0’ (right boxplot) was calculated and the respective BV/TV value was subtracted by BV/TV of T1. Therefore, a value of 0.0 represented a perfect agreement of the corrected/uncorrected scans with those taken without the implant. P values were reported if significant differences were found. Plots were created including the whole available data (*n* = 9). Lower and upper hinges correspond to first and third quartiles, whereas upper and lower whiskers extend to the largest/smallest value no further than 1.5 * IQR (inter-quartile range). Data beyond this range were defined as outliners and plotted as individual points.

When limiting the nine regions to a 2–5 voxel distance, more severe artefacts were observed for all regions (Fig. 3). For these regions, significant differences in BV/TV calculation were found in six out of nine regions.

**Fig. 3:**
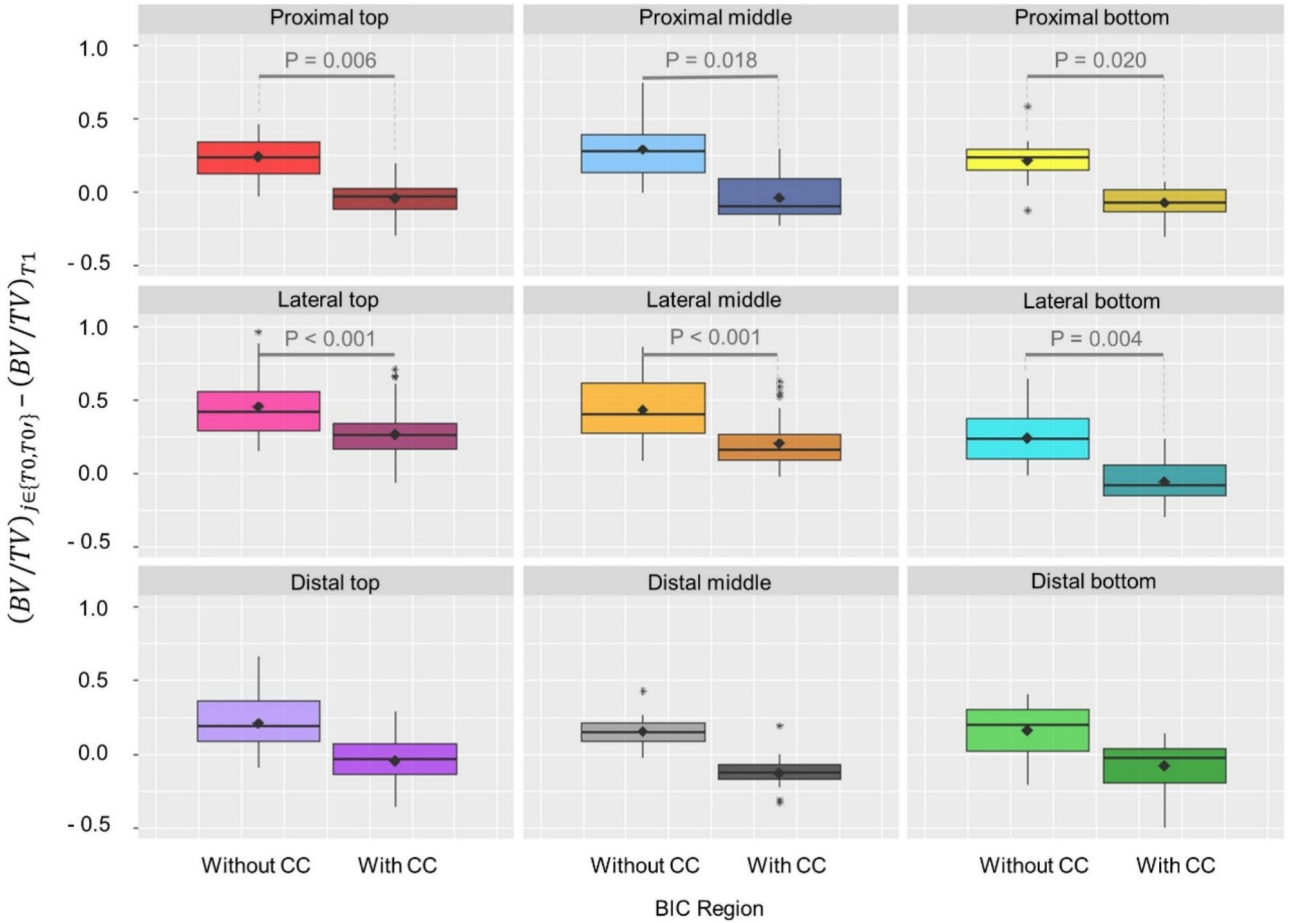
Median BV/TV values + standard deviation and mean values (♦) for the nine segmented peri-implant BIC regions including only the region within 2–5 voxels from the implant. The amount of peri-implant calcified bone volume per total volume BV/TV in T0 (left boxplot) and the corrected T0’ (right boxplot) was calculated and the respective BV/TV value was subtracted by BV/TV of T1. Therefore, a value of 0.0 represents a perfect agreement of scans with and without implant. P values were reported if significant differences were found. Plots were created including the whole available data (*n* = 9). Lower and upper hinges correspond to first and third quartiles, whereas upper and lower whiskers extend to the largest/smallest value no further than 1.5 * IQR (inter-quartile range). Data beyond this range were defined as outliners and plotted as individual points.

### Visual validation

Visual inspection revealed a more evident reduction of metal artefacts on the peri-implant tissue after correction for the scan with implant, compared to the scan without implant (Fig. 4).

**Fig. 4:**
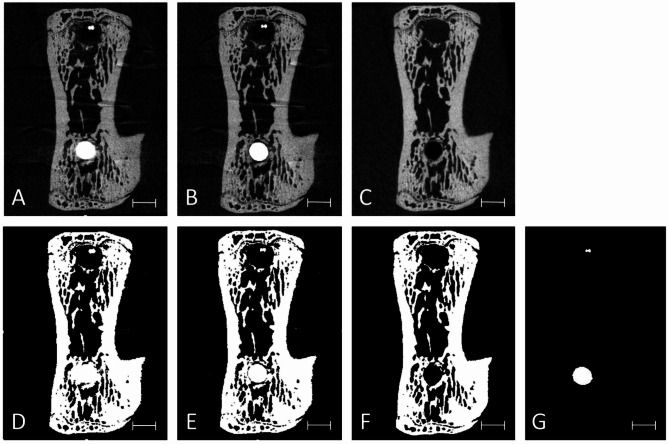
Representative transverse 2D slices of the same animal vertebra depicting (A) T0 scan, (B) $\:{\text{T}}_{0}^{{\prime\:}}$ scan, (C) T1 scan, and (D-F) the segmentation of the respective scans above using a threshold of 29%. Peri-implant over-segmentation due to metal artefacts is visible in the T0 scan (D). Segmentation of Implants using a threshold of 85% in the T0 scan (G). Scale bar equal to 1 mm.

## Discussion

This study aimed to present a regional correction approach to reduce metal artefacts around titanium implants in bone in micro-CT images. Consistent with recent studies^22,23^, a previous study from our group showed that, especially in proximity to the implants, grey values were impeded by metal artefacts and BIC values were systematically overestimated^21^. The present study assumed that grey value alteration would decrease with increasing distance to the titanium implant and therefore, it was expected that a generalized regional correction would enable more accurate segmentation of the peri-implant bone tissue.

The results from the present study confirmed that voxels in close proximity to the implants were much more affected by metal artefacts compared to the more distant ones as described earlier^11,22^. Analysis of the computed correction coefficient revealed a logarithmic relationship between distance to the implant and peri-implant artefacts. By visual inspection of the CC (Fig. 1), the first 100 μm around the implant were most affected, whereas at increasing distance to the implant, the metal artefact induced elevation of grey values became lower. This result is in line with what reported in the canine study of Song et al.^22^ suggesting that a minimum distance of 50 μm from the implant surface is necessary for accurate BIC analyses, since regions closer than 50 μm are severely affected by major artefacts. The specific threshold yielding accurate segmentation may vary not only with distance to the implant, but also depending on implant geometry and material and other factors; however, as shown in Fig. 1, the corrected values were within a reasonable distance to the implant. A significant difference between the mean quotients before and after applying the CC was found. This could infer that, after applying the CC, the grey levels were closer to the grey levels of the dataset without the implant in place (T0). An approximation of the BV/TV values for the peri-implant bone could be achieved. In detail, when analysing the correction across the nine defined peri-implant bone regions, residual artefact values above zero in several regions indicated incomplete correction whereas, overcorrection, marked by values below zero, was observed only in few regions.

In line with previous studies^11,24^, a higher amount of peri-implant tissue was segmented as “bone” when the implant was present and no correction had been applied.

Although the application of the CC largely reduced the amount of artificial bone densification around the implant, the visual inspection and statistical analyses revealed minor differences between the corrected scans ($\:{T}_{0}^{{\prime\:}}$) and the ones without implant (T1), in particular at the implant tip region.

Another factor to be discussed is the original orientation of the scans during the micro-CT imaging. This plays a decisive role regarding where and to what extent metal artefacts occur^8,25,26^. During the scanning process, the implants were parallel to the reconstruction plane (the plane through which the X-rays pass in during one rotation). For this reason, artefacts are to be expected to be more pronounced at the lateral sides of the implant, as well as below its tip. In these regions, the BV/TV values were markly increased compared to the scans without implant, while a lower increase was observed distally and proximally to the implants^7,25,27^. It should be noted that the CC considered only the distance to the implant surface, without accounting for other variables inducing artefacts around the implants (e.g. orientation of the implants in the micro-CT). Consequently, following the application of the CC, BV/TV values remained slightly elevated in regions lateral to the implant in comparison to T1 scans, whereas areas proximal and distal to the implant were slightly overcorrected. However, using the CC a good approximation of BV/TV values was achieved in most cases. However, it has to be noted that, in vertical direction, the top and tip region were excluded since their correction was not requested by the authors, but future studies should also investigate the eligibility of correction in vertical direction outside the implant boundaries.

Further discussion is warranted regarding the additional limitations of this study. First, the appraoch introduced here will only be applicable when the scan set-up and positioning of the implant are very similar, and requires a validation experiment to calculate the correction coefficients. The correction was employing a smoothing spline fit, however, such correction coefficients may also be obtained by a visual determination of the threshold value close to the implant only, and subsequent employment of a global smoothing spline, obtained from different implant geometries and scanning angles. Nonetheless, future studies might investigate if other methods of interpolation or approximation could be even more beneficial. Second, results may remain specific for a particular implant material and size, or require these parameters to be set as a variable. As showed in the systematic review of Terrabuio et al.^28^, the implant material is an essential aspect to be considered when researching metal artefacts. Also the size of the implant has an important effect. The present study was performed on a rat tail vertebra model using implants with a diameter of 0.8 mm. Owing to their small sizes, they most likely did not attenuate the x-rays entirely. For larger implants, the metal artefacts may be more pronounced and the approach used here may not provide satisfying results. In addition, only one implant type was investigated. To generalise the findings future studies are needed with implants of different sizes, geometries and materials. It was also decided to employ a multiplicative correction approach, based on the assumption that applying a weighing factor would facilitate the correction of grey values towards an “average”. In particular, higher values would be given more weight resulting in an adjustement to a greater extent compared to lower values.

Different types of artefacts usually coexist, however in the present study they were not differentiated. Partial volume artefacts are hardly possible to correct owing to the incomplete information of the voxels’ reconstructed grey values. Also Compoton scatter is hard to detect solely by the approach presented here, but owing to the applied filtering, it is likely that Compton scatter had a minor impact on the results. Thus, it is assumed that the methodology presented mainly provided beam hardening correction, which occures due to the fact that a higher amount of photons is absorbed by metal compared to the bone, which eventually hardens the beam in proximity to the implant^28^. Consequentely it is likely that the produced images show artificially high attenuation in the surrounding bone, leading to elevated grey values in the peri-implant region. This phenomenon was particularly observed in the original grey scale images (T0) (Fig. 4A).

It should be noted that the methodology described in the present investigation was specifically developed for studies in the field of micro-CT analysis. Whether artefacts in in vivo dental CT images can be corrected using the same approach remains to be explored, as the nature and extent of the artefacts may vary, and since the fluctuation of grey values among repeated scans is much higher^29^.

To test differences in mean quotients with and without the CC, one could argue that only regions where the CC was applied should be included. However, voxels up to the 65th layer were analysed for differences between $\:{\left(BV/TV\right)}_{j\:\in\:\:\left\{T0,T0{\prime\:}\right\}\:}\--{\left(BV/TV\right)}_{T1}$ using Wilcoxon signed-rank test and each combination of consecutive scans per OMI served as a statistical unit. This approach was specifically designed to demonstrate that, while regions adjacent to the implant are effectively corrected by the CC up to the first 40 voxel layers, the entire sample (extending to layer 65) can be utilized for bone morphometric analyses following application of the correction coefficient to the voxels in proximity to the implant. Nonetheless, the lack of sole application of a correction coefficient just to the voxels in proximity to the implant might be considered as a limitation of the study, since it is known that repeated micro-CT scans have some (minor) fluctuation of grey values, as reported earlier^30^. In the present study, calibration with a phantom was performed as recommended by the manufacturer, ensuring minimal deviation of local grey values. Nevertheless, in proximity to the implant, it is assumed that the fluctuation ca. be higher especially owing to Compton scatter. Further studies are needed to assess the amount of fluctuation in these regions, and to derive optimised scanning parameters to enhance agreement of repeated scans in the presence of titanium implants.

In the present study, analyses revealed no relevant impact of metal artefacts on local grey values within voxel layer 41–65. Therefore, in this area the correction coefficient approximated to 1.0. For the reasons specified earlier, this region was included in the comparison of corrected/uncorrected grey values. Since approximation does not imply equality, it has to be noted that a biasing effect cannot be fully excluded, which constitutes a limitation of the study. However, mathematically speaking, excluding the outer regions from the analyses would represent summation of delta-values that approximate a quasi-constant value since no artefacts were present in this region, and the x-ray machine had been calibrated.

Within the limitations of the study, it was shown that grey value alterations in peri-implant tissues decrease with increasing distance to the implant. This simple estimation of a correction coefficient depending solely on the distance to the implant surface allows for significant improvement of peri-implant grey values. Nevertheless, it should be pointed out that the artefact reduction protocol presented in the present study has been developed and applied on customized orthodontic mini implants made of grade V titanium. Thus, future application might require the recalculation of an adapted CC. This limitation might be overcome in future studies should exploring the the impact of different implant dimensions, geometries and materials.

## Materials and methods

### Animals and study design

A total of *n* = 9 female albino rats of the Wistar strain (mean weight 263 ± 20 g, age 15.4 ± 3.9 weeks) were used. As the present study reports on a subset analysis of a published investigation from our group^21^, no sample size calculation was performed. Indeed, all available ex vivo high-resolution micro-CT scans with and without implants from the aforementioned study were analysed and utilized to compute the CC. An overview of the study design is presented in Suppl. Figure 1. The animals were fed with standard laboratory food pellets and water *ad libitum*. The study protocol was approved by the appropriate local authority (Landesamt für Natur und Verbraucherschutz, Recklinghausen, Germany) (Re. no. 84-02.04.2016.380), the study was performed in accordance with the relevant guidelines and regulations and the reporting conformed to the *Animal Research: Reporting of In Vivo Experiments* (ARRIVE) guidelines 2.0^31^. After an adaptation period of one week, two customized threaded mini-implants (RISystem AG, Landquart, Switzerland) made of a titanium aluminium vanadium alloy, 0.8 mm in diameter and 3.0 mm in length, were placed in the dorsal portion of a single rat tail vertebra and connected with a nickel-titanium contraction spring operating with a randomly assigned loading force of 0 to 1.5 N. Animals utilized within this study were killed at 8 weeks of loading with an overdose of pentobarbital. Detailed information on the study protocol is provided in a previous publication^21^.

### Image acquisition

For each animal, high-resolution micro-CT scans (Viva CT 80, Scanco Medical, Brüttisellen, Switzerland) were performed twice, i.e. immediately after the killing of the animals (T0) and after careful removal of the implants and the nickel-titanium contraction spring (T1). The scans were performed at 70 kVp, 114 µA, and 250 ms integration time and reconstructed to a nominal isotropic voxel size of 10.4 μm. Images were calibrated using a hydroxyapatite phantom.

During scanning, the biopsies were oriented such that the ventro-caudal axis of each vertebra corresponded to the z-axis of the scanner. As the implants were inserted in the dorsal portion of the vertebrae, this implicated that the implant axis was oriented in the XY plane (Suppl. Figure 2). Since the whole tail was positioned within the micro-CT, no other orientation was possible.

### Image processing and registration

Dedicated software, i.e. the Image Processing Language (IPL) as well as the programs uct_evaluation and uct_3d (all from Scanco Medical AG, Brüttisellen, Switzerland), were employed for image processing. The periosteal contour was detected automatically^32^ and used to identify the outer boundaries of the vertebra.

All T0 scans were rotated such that the implants corresponded to the z-axis (Fig. 5). In all cases this alignment was performed for both implants separately, resulting in two datasets for T0, being one co-registered to the proximal and one co-registered to the distal OMI. Thereafter, T1 scans were registered with the T0 reference. During image registration, the region of the implants was excluded as they were only contained in the T0 scans. A Gaussian filter was applied to all images using 0.8 voxel as sigma and 1 voxel as support^33^. Segmentation of the implants and bone was performed by means of thresholds, i.e. 85% and 29% of the possible maximum grey value from all scans, respectively. Furthermore, for implant segmentation, component labelling was employed to remove residual objects other than the implants.

**Fig. 5:**
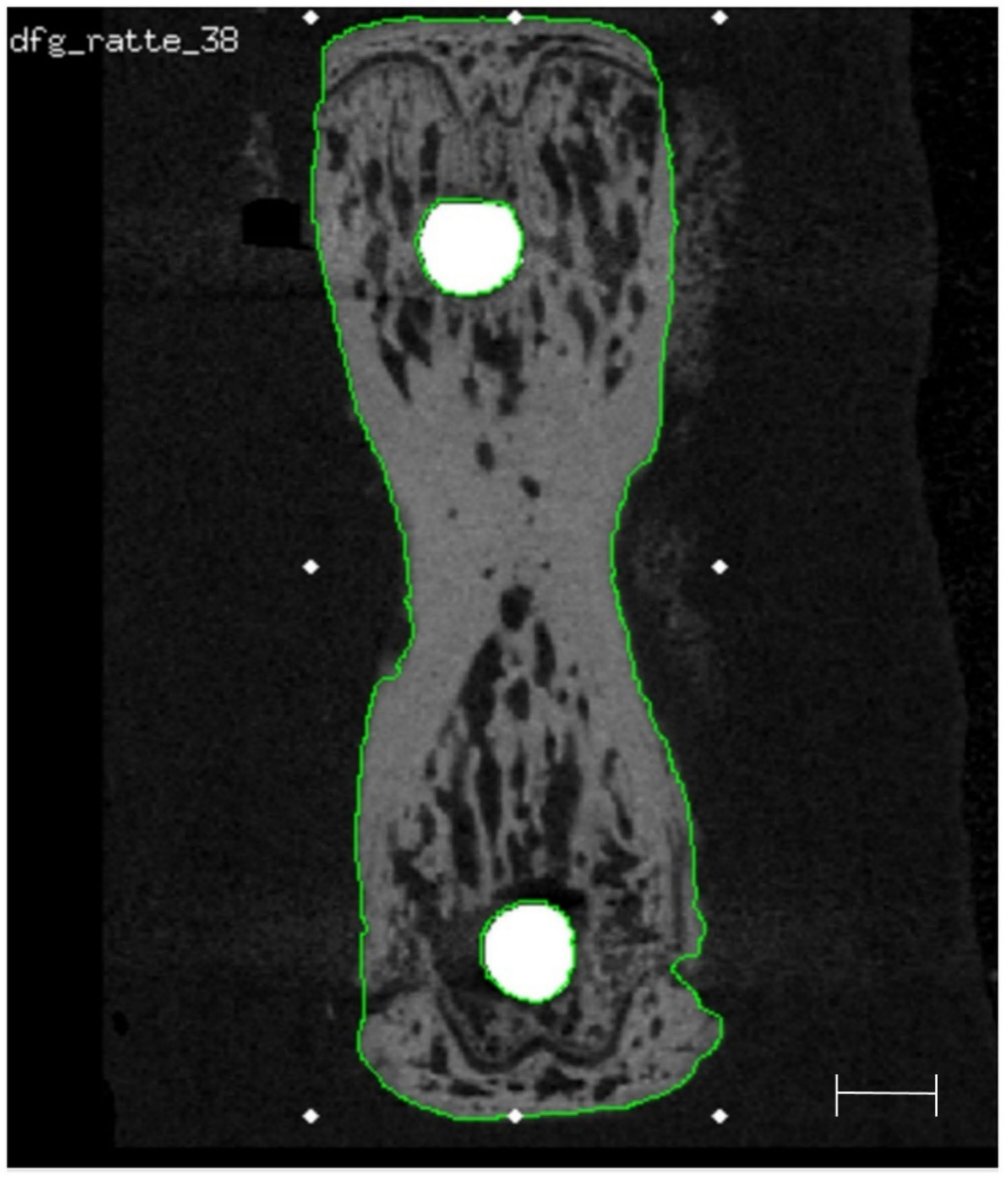
Representative image of a 2D transverse slice of a rat vertebra with implants in situ and the respective VOI used for the registration process (green contour), which excludes the implants (white) and defines the cortical layer and trabecular bone for accurate superimposition of the bone tissue. Scale bar equal to 1 mm.

### Peri-implant grey value comparison

To analyse grey value intensities in direct vicinity to the implants and to assess agreement in different regions later on, twelve standardized cylindric VOIs were determined for each T0 scan and distributed within a corridor of up to 40 voxels (405.6 μm) from the implant. The VOIs had a wall thickness of 2 voxels for the 3 cylinders closest to the implant, of 3 voxels for the next 2 cylinders, and of 4 voxels for the remaining 7 cylinders. To create these standardized VOIs, the implants were first segmented from the images using a high threshold value. Following, the implants were progressively dilated by 2 voxels after which the implant geometry of the previous dilation step was subtracted, thus leaving thin-walled cylindrical masks that were used for identification of the VOIs. As all T1 scans had been registered and transformed at an earlier step, the VOIs created for T0 scans could be applied to the respective T1 scan (Fig. 6).

**Fig. 6:**
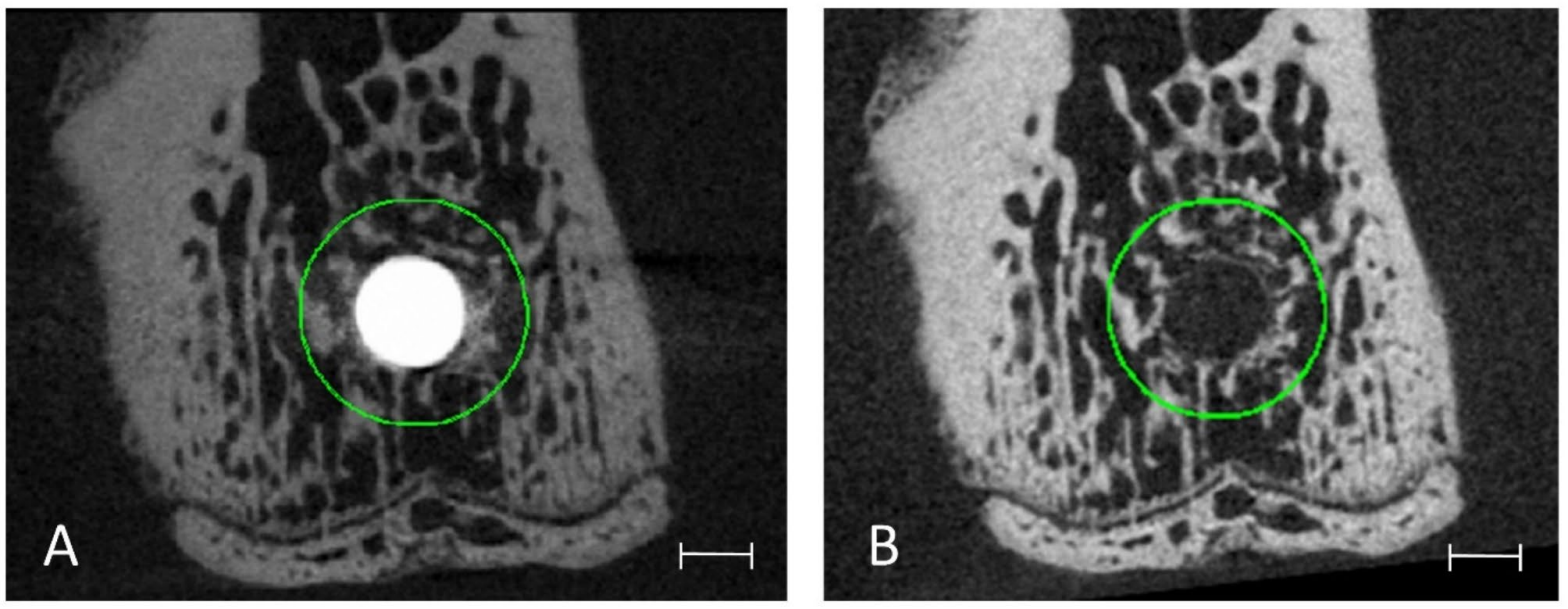
Representative 2D transverse slices of one peri-implant VOI at distance of 39–40 voxels for the T0 scans (A) and its respective T1 scan (B). Note that the actual VOI is a (hollow) cylindrical region, but as it exhibits only 2 voxels of thickness it appears to be a circle. Scale bar equal to 0.5 mm.

### Calculation of the CC

For the calculation of the CC, within the aforementioned 12 cylindrical peri-implant VOIs, the grey values of individual voxels from T1 scans were divided by those of the respective T0 scans, and the mean value per VOI was computed, including grey values along the entire length of the embedded OMI:

1 $$\:{q}_{x}=\sum\:_{i=1}^{n}\left(\frac{{d}_{i,x}^{1}}{{d}_{i,x}^{0}}\right)/n\:$$

where $\:x$ defines the mean Euclidean distance to the implant (in mm), $\:{d}_{i,x}^{T}$ the grey value within the image taken at time point T with T ∈ (0,1) at voxel number $\:i$, $\:i\:\in\:\mathbb{\:}\mathbb{Z}$, *n* the number of voxels in the VOI and $\:{q}_{x}$ the resulting mean quotient within the VOI_x_. Additionally, the respective standard deviations (SD) were assessed per VOI.

Using Matlab’s (The MathWorks Inc., Natick, Massachusetts, United States)^34^ curve fitting toolbox, a smoothing spline *s* (piecewise polynomial consisting of a cubic spline and a least-squares straight-line fit) was computed for the 12 different shells around the implant employing the (automatically specified) smoothing parameter *p* = 0.00023 minimizing the equation:

2 $$\:CC\left(x\right)=\text{m}\text{in}\{p\sum\:_{i}({y}_{i}-s{\left({x}_{i}\right))}^{2}+(1-p)\int\:{\left(\frac{{d}^{2}s}{d{x}^{2}}\right)}^{2}dx\:\}$$

where $\:i$ is the number of observations $\:i\in\:\mathbb{Z}:1<i\le\:12\:$. It must be noted that in Matlab’s curve fitting toolbox, *p* can be defined between 0 and 1. *p* = 0 produces a least-squares straight-line fit to the data, while *p* = 1 produces a cubic spline interpolant. Hence in the present study, a combination of a least-square straight-line fit and a cubic spline interpolant was utilized.

### Application of the CC

$\:CC\left(x\right)\:$was applied to each Voxel $\:{d}_{i,x}^{0}\:$from the T0 images, revealing the corrected $\:{d}_{i,x}^{0{\prime\:}}$ grey value within the corrected T0’ image:

3 $$\:{d}_{i,x}^{0{\prime\:}}={d}_{i,x}^{0}*CC\left(x\right)$$

*and* mean quotients $\:{q}_{x}$ were compared before (4) and after (5) $\:CC\left(x\right)$ was applied in dependence of the distance to the implant, whereby $\:{d}_{i,x}^{\:1}$ denotes grey values from the scan without an implant, and $\:{d}_{i,x}^{\:0}$ and $\:{d}_{i,x}^{\:o{\prime\:}}$ denote grey values of the uncorrected and corrected scans with an OMI.

4 $$\:{q}_{x}=\sum\:_{i=1}^{n}\left(\frac{{d}_{i,x}^{1}}{{d}_{i,x}^{0}}\right)/n\:$$

5 $$\:{q}_{x}^{{\prime\:}}=\sum\:_{i=1}^{n}\left(\frac{{d}_{i,x}^{1}}{{d}_{i,x}^{0{\prime\:}}}\right)/n$$

#### Validation of the CC by BV/TV value comparison

Since the amount of local artefacts in micro-CT scans depends on the orientation of the implant in the micro-CT and the emitted X-ray beams^27^, the authors anticipated that the levels of artefacts around the implants are not equally distributed. Therefore, to quantify the goodness of the correction, the amount of peri-implant calcified bone volume per total volume BV/TV in T0 and the corrected T0’ was calculated to assess the effect of the CC and the respective BV/TV value was subtracted by BV/TV of T1:

6 $$\:{\left(BV/TV\right)}_{j\:\in\:\:\left\{T0,T0{\prime\:}\right\}\:}\--{\left(BV/TV\right)}_{T1}$$

Despite varying amounts of artefacts around the implants were expected owing to the fact that metal artefacts are not equally distributed in all directions around the implant, the CC was solely computed based on the distance to the implant. Thus, it was necessary to conduct analyses within distinct regions around the implant to verify if the CC remained sufficiently effective: nine regions were defined around the implant, i.e. proximal, lateral and distal to the implant, of which each was further divided along the z-axis into top, middle and bottom portions (Fig. 7).

**Fig. 7:**
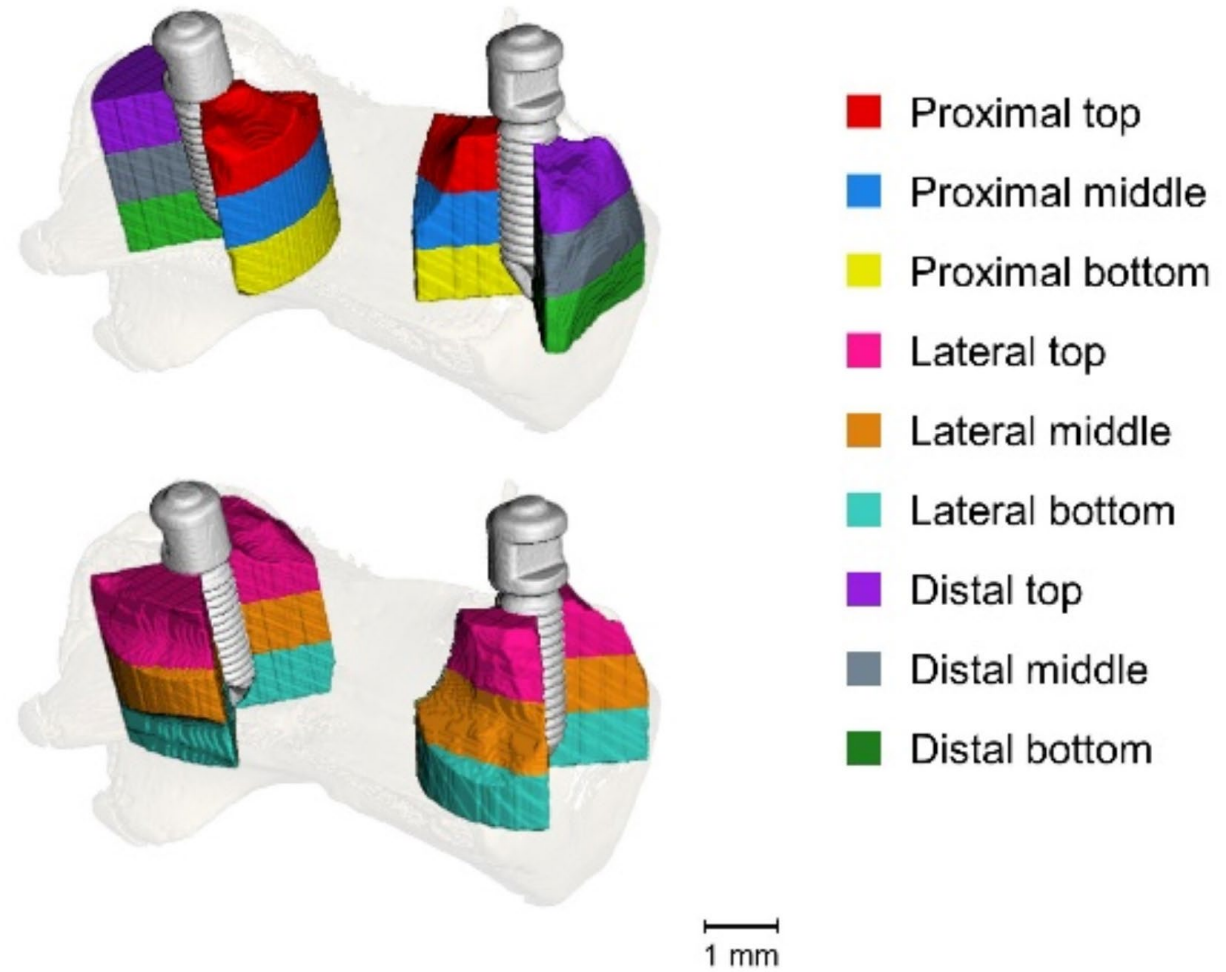
3D rendering of a rat vertebra with the implants with proximal, lateral, and distal peri-implant VOI. The nomenclature for the VOI is presented in the table. From Kerberger et al.^35^.

To reduce the impact of partial volume artefacts, the voxels at the interface with the metal implants were excluded, and the volumes were defined from the second voxel layer onwards. In the first analysis, the regions extended up to 1 mm, corresponding to the 65th voxel layer from the implant (note that the correction was only applied until the 40th voxel layer). Within a second analysis, the regions were limited to the proximity of the implants by including only the region within 2–5 voxels from the implant (BIC region).

### Statistical analysis

The goodness of fit of the smoothing spline fit was analysed in Matlab (The MathWorks Inc.)^36^ by calculating the Root mean square error (RMSE) and the R^2^ values of the fit. A Wilcoxon signed-rank test was performed using R^37^ to test differences between $\:{q}_{x}\:$and $\:{q}_{x}^{{\prime\:}}$ for voxel layers between 2 and 40. Boxplots for $\:{\left(BV/TV\right)}_{j\:\in\:\:\left\{T0,T0{\prime\:}\right\}\:}\--{\left(BV/TV\right)}_{T1}$ were created with the R package ggplot2^38^. For this data, in addition, the differences was evaluated using Wilcoxon signed-rank test for voxel layers between 2 and 65. Each combination of consecutive scans per OMI served as a statistical unit. Results were considered significant for *p* < 0.05.

## Electronic supplementary material

Below is the link to the electronic supplementary material.

Supplementary Information 1.

Supplementary Information 2.

## Data Availability

Data would be available from the corresponding author upon reasonable request.

## References

[1] Feldkamp, L. A., Goldstein, S. A., Parfitt, A. M., Jesion, G. & Kleerekoper, M. The direct examination of three-dimensional bone architecture in vitro by computed tomography. *J. Bone Min. Res.* **4**, 3–11. 10.1002/jbmr.5650040103 (1989). 10.1002/jbmr.5650040103 2718776

[2] Suttapreyasri, S., Suapear, P. & Leepong, N. The accuracy of Cone-Beam computed tomography for evaluating bone density and cortical bone thickness at the implant site: Micro-Computed tomography and histologic analysis. *J. Craniofac. Surg.* **29**, 2026–2031. 10.1097/scs.0000000000004672 (2018). 29894463 10.1097/SCS.0000000000004672

[3] Rüegsegger, P., Koller, B. & Müller, R. A microtomographic system for the nondestructive evaluation of bone architecture. *Calcif Tissue Int.* **58**, 24–29. 10.1007/bf02509542 (1996). 8825235 10.1007/BF02509542

[4] Becker, K., Stauber, M., Schwarz, F. & Beißbarth, T. Automated 3D-2D registration of X-ray microcomputed tomography with histological sections for dental implants in bone using chamfer matching and simulated annealing. *Comput. Med. Imaging Graph*. **44**, 62–68. 10.1016/j.compmedimag.2015.04.005 (2015). 26026659 10.1016/j.compmedimag.2015.04.005

[5] Stadelmann, V. A., Conway, C. M. & Boyd, S. K. In vivo monitoring of bone-implant bond strength by MicroCT and finite element modelling. *Comput. Methods Biomech. Biomed. Engin*. **16**, 993–1001. 10.1080/10255842.2011.648625 (2013). 22289208 10.1080/10255842.2011.648625

[6] Draenert, F. G., Coppenrath, E., Herzog, P., Müller, S. & Mueller-Lisse, U. G. Beam hardening artefacts occur in dental implant scans with the NewTom cone beam CT but not with the dental 4-row multidetector CT. *Dentomaxillofac Radiol.* **36**, 198–203. 10.1259/dmfr/32579161 (2007). 17536086 10.1259/dmfr/32579161

[7] Min, C. K. & Kim, K. A. Quantitative analysis of metal artefacts of dental implant in CBCT image by correlation analysis to micro-CT: A microstructural study. *Dentomaxillofac Radiol.* **50**, 20200365. 10.1259/dmfr.20200365 (2021). 33002369 10.1259/dmfr.20200365 PMC7923071

[8] Li, J. Y. et al. Quantitative analysis of titanium-induced artifacts and correlated factors during micro-CT scanning. *Clin. Oral Implants Res.* **25**, 506–510. 10.1111/clr.12200 (2014). 23745988 10.1111/clr.12200

[9] Wanderley, V. A. et al. Impact of the blooming artefact on dental implant dimensions in 13 cone-beam computed tomography devices. *Int. J. Implant Dent.* **7**, 67. 10.1186/s40729-021-00347-6 (2021). 34258634 10.1186/s40729-021-00347-6 PMC8277908

[10] Schulze, R. et al. Artefacts in CBCT: a review. *Dentomaxillofac Radiol.* **40**, 265–273. 10.1259/dmfr/30642039 (2011). 21697151 10.1259/dmfr/30642039 PMC3520262

[11] Bissinger, O. et al. Comparative 3D micro-CT and 2D histomorphometry analysis of dental implant osseointegration in the maxilla of minipigs. *J. Clin. Periodontol*. **44**, 418–427. 10.1111/jcpe.12693 (2017). 28063250 10.1111/jcpe.12693

[12] Abbassy, M. A. Fluoride influences nickel-titanium orthodontic wires’ surface texture and friction resistance. *J. Orthod. Sci.* **5**, 121–126. 10.4103/2278-0203.192114 (2016). 27843886 10.4103/2278-0203.192114 PMC5084473

[13] Schouten, C., Meijer, G. J., van den Beucken, J. J., Spauwen, P. H. & Jansen, J. A. The quantitative assessment of peri-implant bone responses using histomorphometry and micro-computed tomography. *Biomaterials* **30**, 4539–4549. 10.1016/j.biomaterials.2009.05.017 (2009). 19500840 10.1016/j.biomaterials.2009.05.017

[14] Mahnken, A. H. et al. A new algorithm for metal artifact reduction in computed tomography: in vitro and in vivo evaluation after total hip replacement. *Invest. Radiol.* **38**, 769–775. 10.1097/01.rli.0000086495.96457.54 (2003). 14627894 10.1097/01.rli.0000086495.96457.54

[15] Huflage, H. et al. Metal artefact reduction in low-dose computed tomography: benefits of Tin prefiltration versus postprocessing of dual-energy datasets over conventional CT imaging. *Radiography (Lond)*. **28**, 690–696. 10.1016/j.radi.2022.05.006 (2022). 35728278 10.1016/j.radi.2022.05.006

[16] Cheraya, G., Sharma, S. & Chhabra, A. Dual energy CT in musculoskeletal applications beyond crystal imaging: bone marrow maps and metal artifact reduction. *Skeletal Radiol.* **51**, 1521–1534. 10.1007/s00256-021-03979-2 (2022). 35112139 10.1007/s00256-021-03979-2

[17] Schmitt, N. et al. The impact of software-based metal artifact reduction on the liquid embolic agent Onyx in cone-beam CT: a systematic in vitro and in vivo study. *J. Neurointerv Surg.* **14**, 832–836. 10.1136/neurintsurg-2021-018018 (2022). 34433643 10.1136/neurintsurg-2021-018018 PMC9304113

[18] Niehues, S. M., Vahldiek, J. L., Tröltzsch, D., Hamm, B. & Shnayien, S. Impact of Single-Energy metal artifact reduction on CT image quality in patients with dental hardware. *Comput. Biol. Med.* **103**, 161–166. 10.1016/j.compbiomed.2018.10.023 (2018). 30384174 10.1016/j.compbiomed.2018.10.023

[19] Katsura, M., Sato, J., Akahane, M., Kunimatsu, A. & Abe, O. Current and novel techniques for metal artifact reduction at CT: practical guide for radiologists. *Radiographics* **38**, 450–461. 10.1148/rg.2018170102 (2018). 29528826 10.1148/rg.2018170102

[20] Wellenberg, R. H. H. et al. Metal artifact reduction techniques in musculoskeletal CT-imaging. *Eur. J. Radiol.* **107**, 60–69. 10.1016/j.ejrad.2018.08.010 (2018). 30292274 10.1016/j.ejrad.2018.08.010

[21] Becker, K. et al. Can implants move in bone? A longitudinal in vivo micro-CT analysis of implants under constant forces in rat vertebrae. *Clin. Oral Implants Res.* **30**, 1179–1189. 10.1111/clr.13531 (2019). 31494964 10.1111/clr.13531

[22] Song, J. W., Cha, J. Y., Bechtold, T. E. & Park, Y. C. Influence of peri-implant artifacts on bone morphometric analysis with micro-computed tomography. *Int. J. Oral Maxillofac. Implants*. **28**, 519–525. 10.11607/jomi.1632 (2013). 23527354 10.11607/jomi.1632

[23] Stoppie, N. et al. Validation of microfocus computed tomography in the evaluation of bone implant specimens. *Clin. Implant Dent. Relat. Res.* **7**, 87–94. 10.1111/j.1708-8208.2005.tb00051.x (2005). 15996355 10.1111/j.1708-8208.2005.tb00051.x

[24] Bernhardt, R. et al. Comparison of microfocus- and synchrotron X-ray tomography for the analysis of osteointegration around Ti6Al4V implants. *Eur. Cell Mater.* **7**, 42–51; discussion 51 (2004). 10.22203/ecm.v007a05 10.22203/ecm.v007a05 15375777

[25] Luckow, M., Deyhle, H., Beckmann, F., Dagassan-Berndt, D. & Müller, B. Tilting the jaw to improve the image quality or to reduce the dose in cone-beam computed tomography. *Eur. J. Radiol.* **80**, e389–393. 10.1016/j.ejrad.2010.10.001 (2011). 21035293 10.1016/j.ejrad.2010.10.001

[26] Lewis, M., Toms, A. P., Reid, K. & Bugg, W. CT metal artefact reduction of total knee prostheses using Angled gantry multiplanar reformation. *Knee* **17**, 279–282. 10.1016/j.knee.2010.02.007 (2010). 20356751 10.1016/j.knee.2010.02.007

[27] Min, C. K. & Kim, K. A. Reducing metal artifacts between implants in cone-beam CT by adjusting angular position of the subject. *Oral Radiol.* **37**, 385–394. 10.1007/s11282-020-00458-7 (2021). 32638201 10.1007/s11282-020-00458-7

[28] Terrabuio, B. R. et al. Cone-beam computed tomography artifacts in the presence of dental implants and associated factors: an integrative review. *Imaging Sci. Dent.* **51**, 93–106. 10.5624/isd.20200320 (2021). 34235055 10.5624/isd.20200320 PMC8219451

[29] Nackaerts, O. et al. Analysis of intensity variability in multislice and cone beam computed tomography. *Clin. Oral Implants Res.* **22**, 873–879. 10.1111/j.1600-0501.2010.02076.x (2011). 21244502 10.1111/j.1600-0501.2010.02076.x

[30] Kim, Y., Brodt, M. D., Tang, S. Y. & Silva, M. J. MicroCT for scanning and analysis of mouse bones. *Methods Mol. Biol.* **2230**, 169–198. 10.1007/978-1-0716-1028-2_11 (2021). 33197015 10.1007/978-1-0716-1028-2_11 PMC8409170

[31] du Percie, N. et al. The ARRIVE guidelines 2.0: updated guidelines for reporting animal research. *J. Physiol.* **598**, 3793–3801. 10.1113/jp280389 (2020). 32666574 10.1113/JP280389 PMC7610696

[32] Buie, H. R., Campbell, G. M., Klinck, R. J., MacNeil, J. A. & Boyd, S. K. Automatic segmentation of cortical and trabecular compartments based on a dual threshold technique for in vivo micro-CT bone analysis. *Bone* **41**, 505–515. 10.1016/j.bone.2007.07.007 (2007). 17693147 10.1016/j.bone.2007.07.007

[33] Campbell, G. M. & Sophocleous, A. Quantitative analysis of bone and soft tissue by micro-computed tomography: applications to ex vivo and in vivo studies. *Bonekey Rep.* **3**, 564. 10.1038/bonekey.2014.59 (2014). 25184037 10.1038/bonekey.2014.59 PMC4140449

[34] The MathWorks Inc. *Curve Fitting Toolbox*. (2021). https://de.mathworks.com/products/curvefitting.html

[35] Kerberger, R., Brunello, G., Drescher, D., van Rietbergen, B. & Becker, K. Micro finite element analysis of continuously loaded mini-implants - A micro-CT study in the rat tail model. *Bone* **177**, 116912. 10.1016/j.bone.2023.116912 (2023). 37739299 10.1016/j.bone.2023.116912

[36] The MathWorks Inc. *Curve Fitting Toolbox; Evaluating Goodness of Fit*. (2021). https://de.mathworks.com/help/curvefit/evaluating-goodness-of-fit.html

[37] R Core Team. *R: A Language and Environment for Statistical Computing. R Foundation for Statistical Computing.* (2024). https://www.R-project.org/

[38] Wickham, H. *ggplot2: Elegant Graphics for Data Analysis* (Springer, 2016).

