# Supplementary figures and images for: Correction of metal artefacts around orthodontic mini-implants – a micro-CT study in the rat tail model
Source: Sci Rep. 2025 Apr 1;15:11133. doi: 10.1038/s41598-025-93411-9 (PMC11962067; doi:10.1038/s41598-025-93411-9)

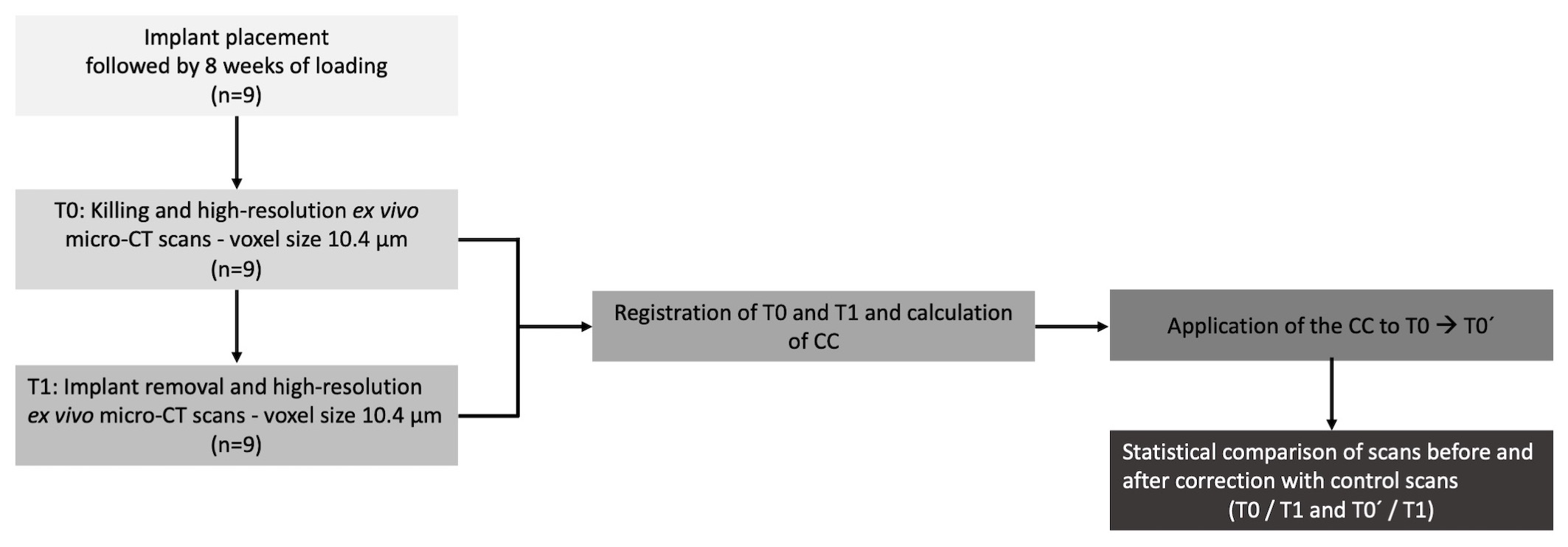

Supplement: Supplementary file 1 — Supplementary Information 1. [file 41598_2025_93411_MOESM1_ESM.jpeg]

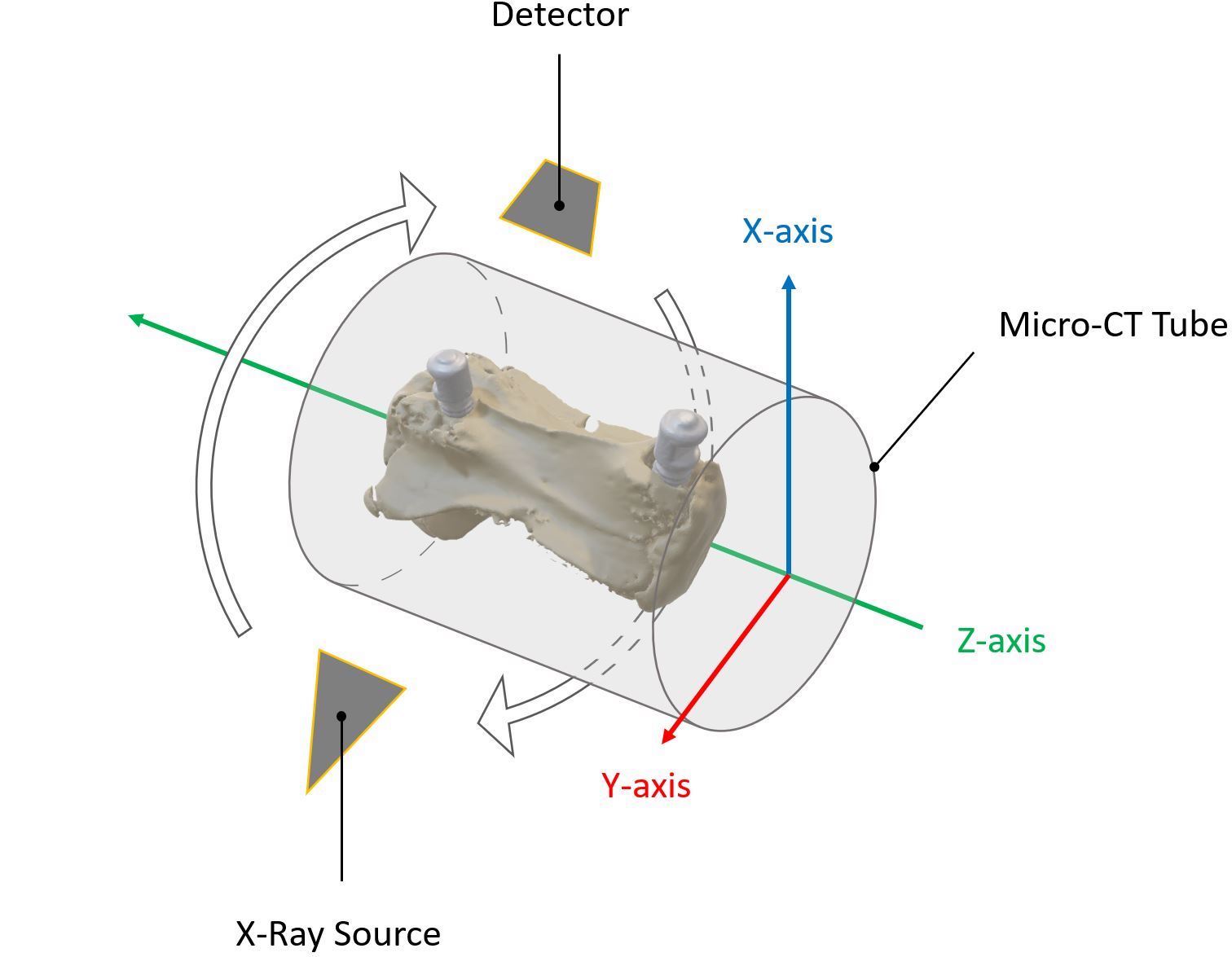

Supplement: Supplementary file 2 — Supplementary Information 2. [file 41598_2025_93411_MOESM2_ESM.jpg]
